# Minimal invasive extracorporeal circulation an alternative to ECMO in ventricular tachycardia ablation

**DOI:** 10.1051/ject/2023031

**Published:** 2023-09-08

**Authors:** Ignazio Condello

**Affiliations:** 1 Department of Cardiac Surgery, GVM Care & Research, Perfusion Service, Anthea Hospital Via Camillo Rosalba 35/37 70124 Bari Italy

**Keywords:** Minimally invasive extra-corporeal circulation (MiECC), Ablation, Ventricular tachycardia, Extracorporeal membrane oxygenation (ECMO)

## Abstract

*Background*: The advantages of mechanical assistance during ventricular tachycardia (VT) ablation have not been clinically demonstrated. We propose and discuss a technique, set up by us, that makes use of minimally invasive extra-corporeal circulation (MiECC) type III associated with a venous reservoir system, which allows complete cardiac flow support and blood oxygenation as well as hemodynamic stability during long-lasting procedures. *Methods*: We present a retrospective case series of ten patients with valvular heart disease and unresponsive Ventricular Tachycardia (VT) who underwent VT ablation with MiECC support. The mean age of the patients was 72 ± 8 years and the left ventricular ejection fraction was 36 ± 12%. All patients underwent a clinical evaluation to identify the cause of VT unresponsiveness (e.g., ischemic heart disease). *Results*: A total of 140 min, the following parameters were evaluated and recorded for 140 min. Central venous pressure (CVP) was used to evaluate excess volume. During the first 5 min, the mean was 15 mmHg, with a pump flow of 1.5 L/min and a mean systemic arterial pressure of 100 mmHg while setting up the circulation support. Following drainage in a volumetric bag of 1 L of blood, CVP was reduced to a value of 5 mmHg with a flow rate of 5 L/min and a mean systemic arterial pressure of 65 mmHg. In the case of small and low-weight patients our “1 L protocol” can be modified. *Conclusions*: In this preliminary retrospective case series, the MiECC type III system may represent the ideal support system during VT ablation, and further studies are needed to support this preliminary report.

## Background

Catheter ablation is an elective therapeutic strategy for the treatment of ventricular tachycardia (VT) in structural heart diseases with and without comorbidities [1]. Clinically, complete VT ablation involves a long-lasting procedure with extensive activation and substrate mapping to localize the circuit isthmus to improve procedural success and minimize follow-up complications. However, >50% of patients with structural heart disease requiring VT ablation are hemodynamically unstable, indicating the need for strict clinical assessment of intraoperative hemodynamic support [2]. In general, hemodynamic support should ensure that adequate blood pressure levels and end-organ perfusion are maintained, thus reducing the risk of periprocedural heart failure and favoring rapid postintervention recovery. Although preliminary reports do not show a clear advantage of percutaneous ventricular support devices [3], their use during VT ablation has recently received renewed interest [3]. Indeed, decision-makers should consider that hemodynamic support devices can be of further help in the case of a learning curve, with operators performing these procedures that need “physiologically” longer times to complete the procedure itself. In our experience, although the procedures are performed by experienced electrophysiologists, the need for more than six ablations due to the presence of multiple ventricular arrhythmic foci usually leads to procedures lasting up to 3 h.

Historically, passive intra-aortic balloon counter pulsation has been the most frequently used hemodynamic support has been the passive intra-aortic balloon counter pulsation [4]. However, intra-aortic balloon counter pulsation provides a very limited increase of cardiac output (only 0.5 L/min) [5], equipment with active pump action has found more large applications during complex hemodynamic procedures [4].

More recently, extracorporeal membrane oxygenation (ECMO) has emerged as a valid alternative support method in clinical practice [4]. In contrast to the aforementioned hemodynamic support devices, ECMO, in addition, to pump support, oxygenates the patient’s blood, ensuring a more comprehensive metabolic control of the cardiac tissue [6]. The limitation of this circuit lies in its “closed architecture,” which makes managing blood volume impossible, especially if intra-procedural complications occur and/or rapid volume replacement or emptying of the heart is needed. Appropriate management of the blood volume is essential to ensure adequate cardiac output, preserve tissue metabolism, and reduce mechanical tension on the ventricular wall. A potential role has recently, minimally invasive extracorporeal circulation (MiECC) assistance during invasive procedures has been recognized [7]. The MiECC circuit consists of a centrifugal pump with a membrane oxygenator, short heparin-coated tubing with biologically inert surfaces, a heat exchanger, venous de-airing components, and a shed-blood management system that minimizes the pathophysiological side effects of conventional extracorporeal circulation [8]. Nevertheless, data regarding the prevalence, predictors, and clinical outcomes of patients receiving hemodynamic support during VT ablation remain limited and sparse [2].

This pilot study aimed to report our experience with endocardial catheter ablation of VT supported by a ventricular assistance system with the possibility of simultaneously oxygenating the blood and managing the blood volume. We describe a special use of MiECC type III, which preserves the main principles of the minimally invasive extra-corporeal technology, such as a closed extra-corporeal circulation circuit, biologically inert blood contact surfaces, and reduced priming volume while maintaining the equilibrium and the ability to manage excessive volume in a closed extra-corporeal circuit [9].

## Materials and methods

We present a case series of ten patients with valvular heart disease and unresponsive VT who underwent VT ablation with MiECC support. The mean age of the patients was 72 ± 8 years and the left ventricular ejection fraction was 36 ± 12% . The patient characteristics are shown in Table 1. All patients underwent a clinical evaluation to identify the cause of VT unresponsiveness (e.g., ischemic heart disease). However, neither significant valvular heart disease nor ischemic heart disease was observed. The MiECC III strategy was used to better manage blood volume, especially in patients with concomitant valvular heart disease that was not surgically treated (e.g., a patient with associated severe aortic valve stenosis with risk of ventricular dilatation and severe mitral insufficiency).

**Table 1:** Patient data.

Procedures	All ventricular tachycardia ablation (*n* = 10)
Age (years)	72 ± 8
Male sex	4
Left ventricular ejection fraction (%)	36 ± 12
Mitral valve stenosis (nr)	2
Aortic valve insufficiency (nr)	2
Without valve disease	6
EuroSCORE II (mean)	3.6

Values are presented as *n* (%) or mean ± standard deviation.

EuroSCORE: European System for Cardiac Operative Risk Evaluation.

**Figure 1:**
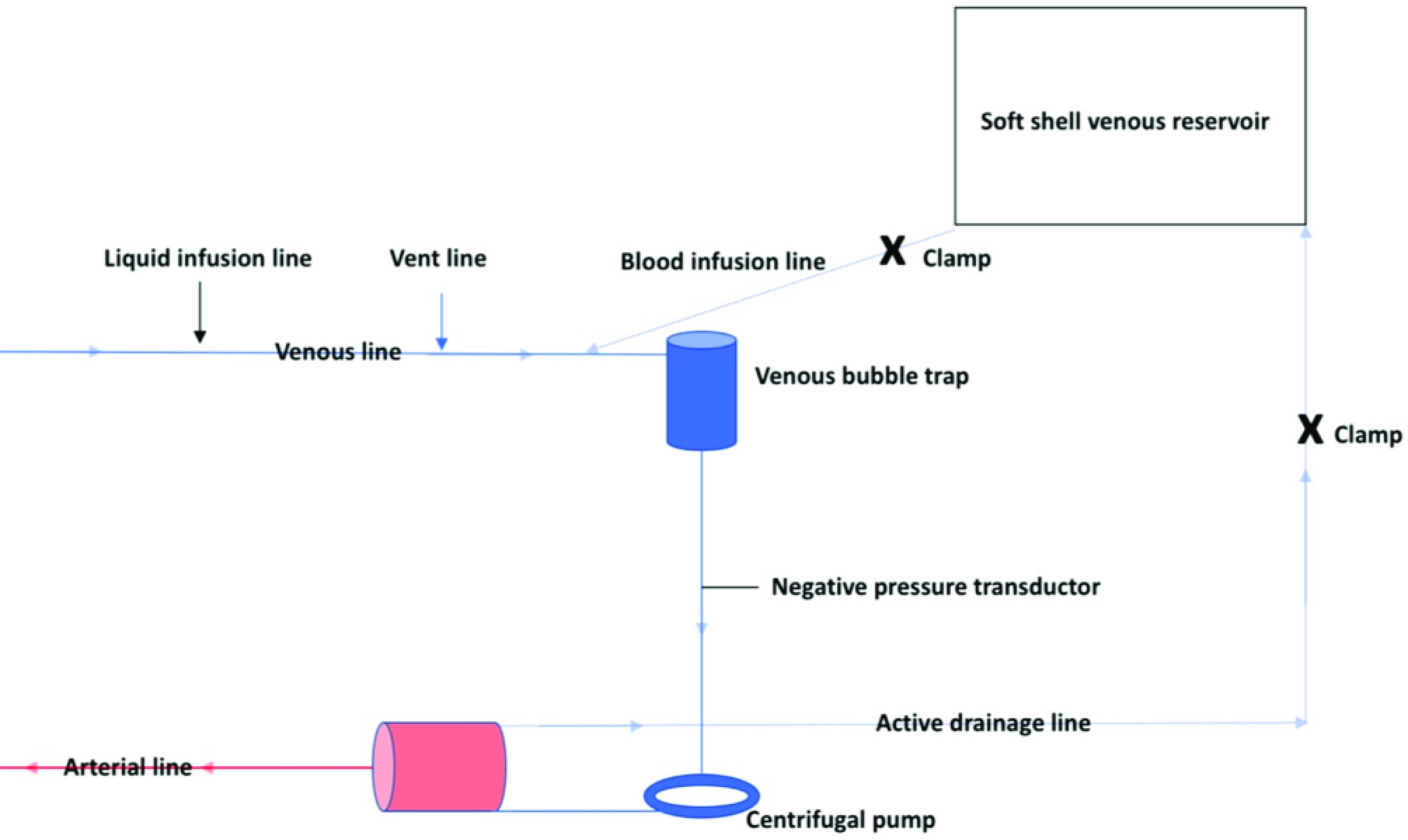
Minimally invasive extra-corporeal circulation (MiECC) type III – sketch.

**Figure 2:**
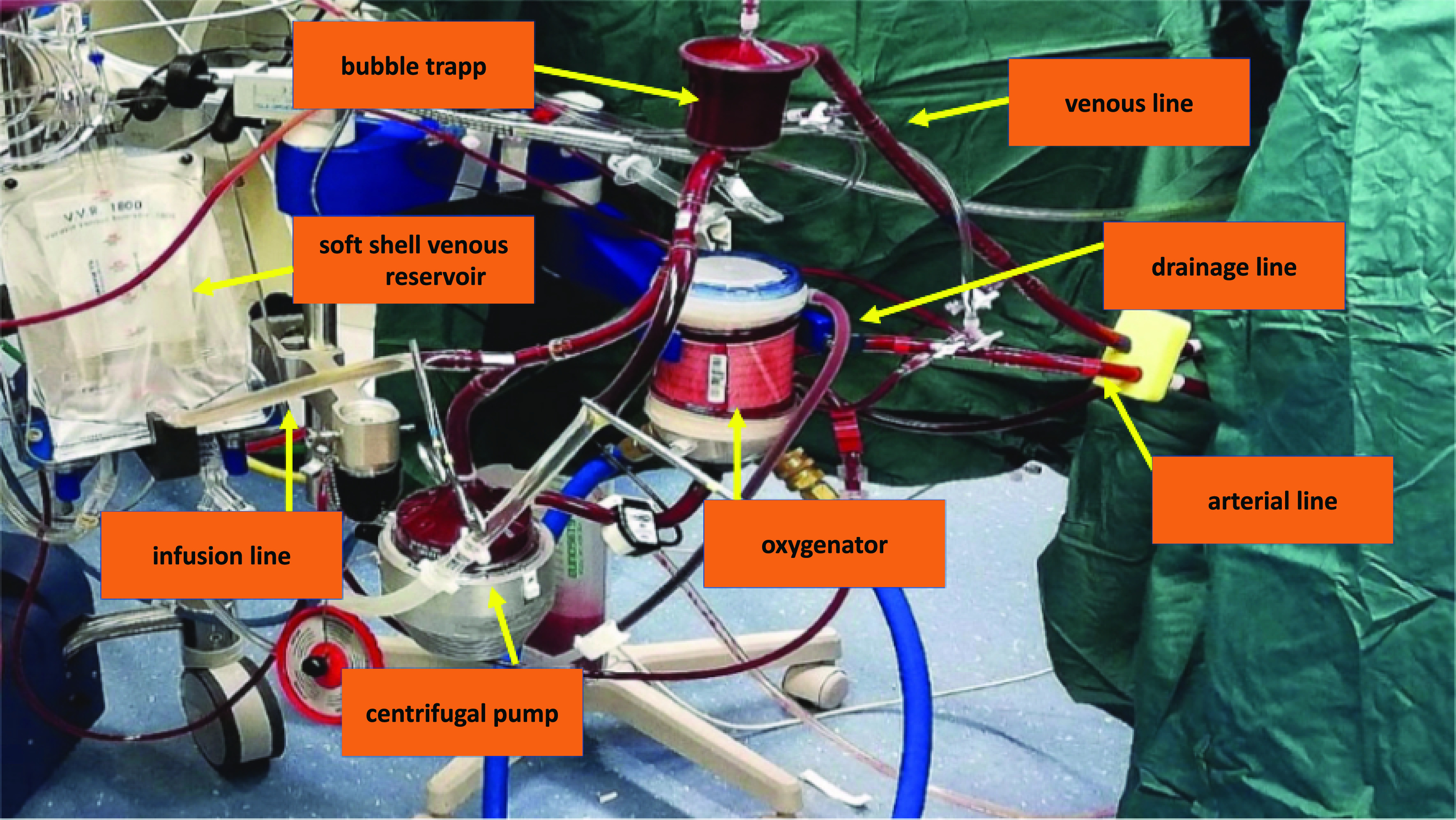
Minimally invasive extra-corporeal circulation (MiECC) type III – description of the components.

**Figure 3:**
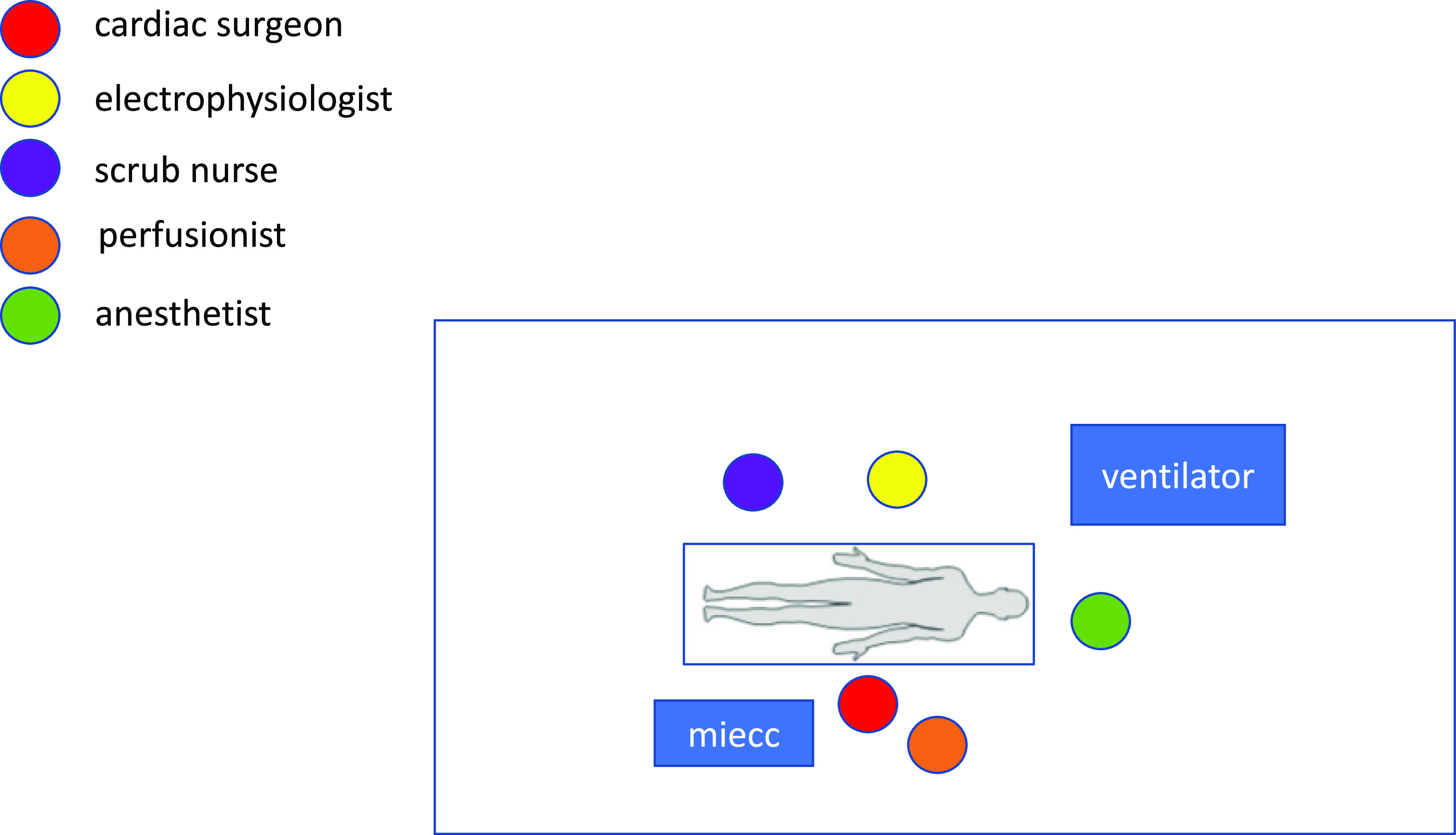
Organization and position of the Ablation and MiECC teams in relation to the patient – sketch.

More specifically, we modified the classical ECMO circuit by adding a volumetric bag for blood volume management (Figures 1 and 2). Cannulation was performed through a mini-incision of the left groin, and after heparin administration, the cannulas were placed under direct vision with sutures in 5/0 polypropylene. Catheters for mapping and ablation were positioned by percutaneous puncture of the right groin (Figure 3). These procedures can be performed either in the catheterization lab or in a hybrid room.

The internal research board of our institution, Anthea Hospital, approved this study (Prot. 01/2020), and all patients provided their consent for the procedure and the use of data.

## Results

**Figure 4:**
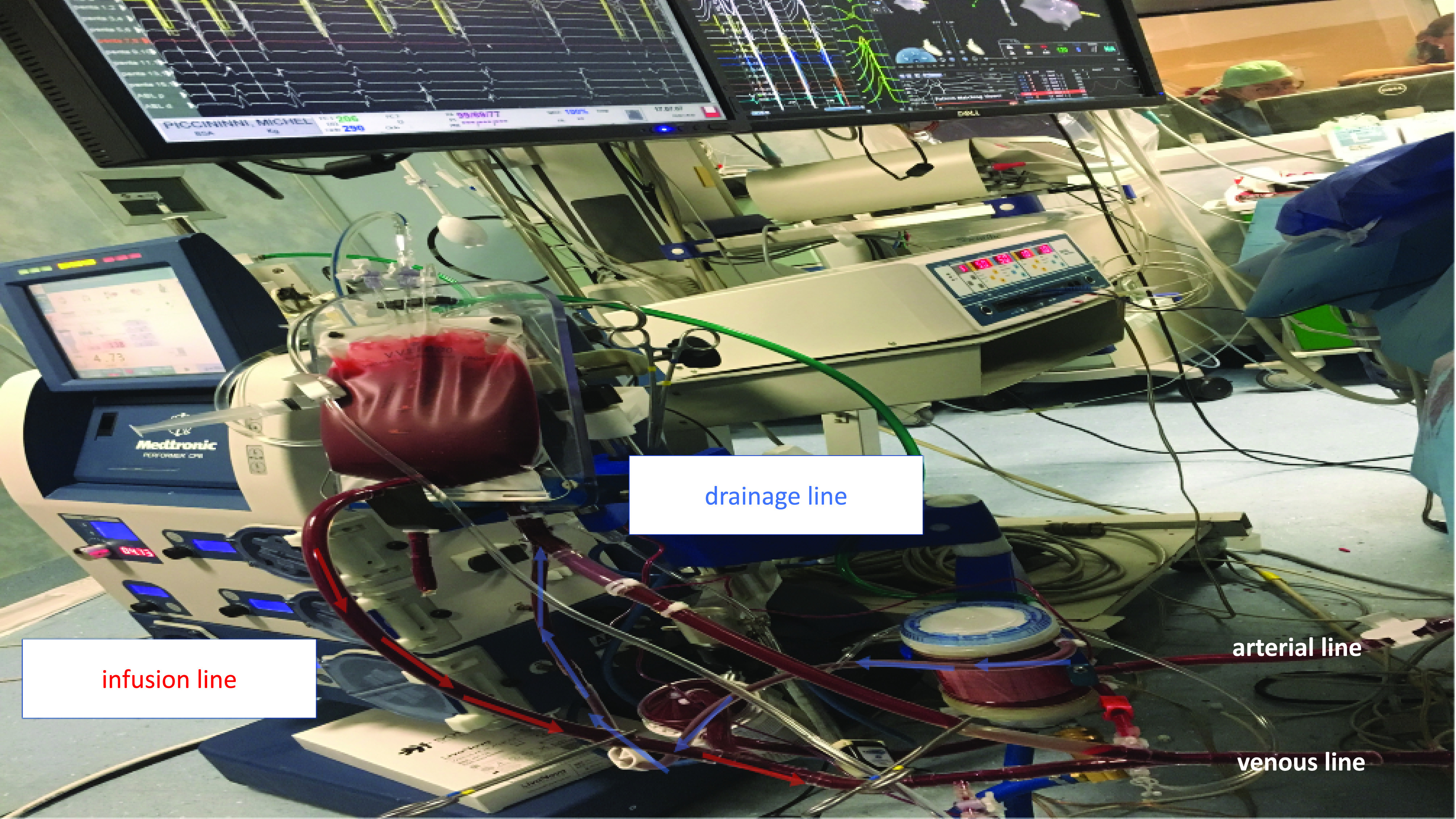
Minimally invasive extra-corporeal circulation (MiECC) type III – during the procedure.

In our practice, a double team experienced in ablation and MiECC performed these procedures [4]. The “key” concept is: to “replicate” concomitantly the two procedures in parallel, for the cardiac surgery team the MiECC, and for the electrophysiology team ablation. During the establishment of circulation, for a total of 140 min, the following parameters were evaluated and recorded:

In the first 3 min, volume overload causes flow reduction and left ventricular afterload excess. Since afterload excess might overload the left ventricle (given the presence of aortic regurgitation), to reduce wall tension, excess fluid was actively drained from the outlet of the oxygenator in the volumetric bag (Figure 4).

Central venous pressure (CVP) was used to evaluate excess volume. For example, during the first 5 min, the mean was 15 mmHg, with a pump flow of 1.5 L/min and a mean systemic arterial pressure of 100 mmHg while setting up the circulation support. Following drainage in a volumetric bag of 1 L of blood, CVP was reduced to a value of 5 mmHg with a flow rate of 5 L/min and a mean systemic arterial pressure of 65 mmHg. In the case of small and low-weight patients our “1 L protocol” can be modified.

The volume was subsequently distributed gradually in the venous line near the centrifugal pump during the procedure using the antihypertensive drug Ebrantil (usually a 10 mg bolus) that allowed immediate volume redistribution without excessive hypotension [10], in the absence of venous stasis. Decannulation, as the cannulation in our protocol, involves surgical exposure.

Volume management in MiECC type III, in VT ablation procedures with and without the presence of heart valve disease, with mean Left ventricular ejection fraction (%) 36 ± 12 allowed in the procedures a better drainage from a central venous pressure (CVP) of 15 ± 4 to a CVP of 4 ± 3, an improvement in relation to flow rate of the pump from 1.5 ± 0.6 L/min to 5 ± 0.7 L/min, in relation to mean arterial pressure (MAP) from 100 ± 18 to 65 ± 13 mmHg, with an mean hemoglobin value of 9.78 ± 1.19 g/dL Table 2.

**Table 2:** Pre- and intra-procedural data.

Procedures	Ventricular tachycardia ablation (*n* = 10)
Pre-CPB hematocrit (%)	36.7 ± 1.9
Pre-CPB Hb (g/dL)	11.9 ± 1.8
CPB time (min)	140 ± 18
Perioperative CVP target (mmHg) on MiECC without Volume management	15 ± 4
Perioperative ACT (s)	400 ± 60
Hb (g/dL) during CPB	9.78 ± 1.19
Mean arterial pressure (mmHg) before volume management	100 ± 18
Blood flow (L/min) before volume management	1.5 ± 0.6
Mean arterial pressure (mmHg) after volume management	65 ± 13
CVP target (mmHg) after volume management in MiECC	4 ± 3
Blood flow (L/min) after volume management	5 ± 0.7

Values are presented as *n* (%) or mean ± standard deviation.

CVP: Central Venous Pressure; CPB: cardiopulmonary bypass; Hb: hemoglobin; MiECC: minimally invasive extracorporeal circulation.

### Statistical analysis

Continuous data are expressed as mean ± standard deviation or median with interquartile range, and categorical data as percentages. Cumulative survival was evaluated using the Kaplan–Meier method.

## Discussion

Based on our experience, the insertion of a soft-shell venous reservoir in the MiECC type III system improved volume management in patients with large surfaces and volumes, reduced afterload [11], and improved pump flow, as observed in patients suffering from arrhythmias associated with valvular heart disease.

The circuits that we used in this context for MiECC are usually employed for conventional extracorporeal circulation with a polypropylene oxygenator (maximum duration of 6 h). This avoids the use of devices (e.g., oxygenators) specifically designed for ECMO and for prolonged use (several days if the oxygenator is made of PMP). VT ablation is a short-term procedure, and conventional materials can be used with subsequent economic advantages. In patients with a high bleeding risk, it may be convenient to use “prolonged” ECMO circuits, which require a lower activated clotting time (ACT) to ensure less bleeding risk. However, in the case of ACT >400 s (and/or <400 s), concomitantly with inguinal preparation for contralateral groin cannulation, we deemed it necessary to place the introducers for the subsequent mapping and ablation catheters before heparin administration to facilitate puncture and reduce the risk of hematoma.

Another advantage of our modified MiECC system is the presence of a heat exchanger that heats the patient’s blood as the ECMO system. This system, which maintains the patient’s temperature despite the prolonged procedure not only facilitates hemodynamic and coagulation stability but also allows direct patient extubation soon after the end of the procedure, so that the patient does not need to stay in an intensive care unit.

We agree with Virk et al. [4] that the benefits of using ventricular assist device systems during VT ablation may be established only through randomized trials, but we also believe that, to date, the benefits of this hemodynamic support have not been recorded because the support systems used are inadequate for this purpose. Most of the previous studies reported results obtained with a “partial” circulation support with the Impella system [3]. This has obviously impacted the results summarized in the meta-analysis by Turagam et al. [3], who reported that patients treated with support systems do not benefit from hemodynamic support but experience more complications [3]. The poor pumping and oxygenation capacity makes the aforementioned systems (when the heart has no activity because “immobilized” by the ablation maneuvers, the blood does not move, nor can it be oxygenated by the lungs) inadequate. Therefore, the most effective system is ECMO. Simultaneously, ECMO does not allow the operator to completely empty the heart that remains filled, particularly if untreated valvulopathies coexist (e.g., aortic insufficiency), ultimately leading to ischemic complications and acute ventricular failure. Aortic insufficiency could be a contraindication to peripheral ECMO support due to the distension of the left ventricle, which involves an increase in the subendocardial wall tension, not favoring perfusion of the microcirculation and myocardial recovery; thus, venting of the left ventricle is required. Furthermore, an empty heart, especially if endocardial ablation should be performed but for better visualization, also in case of epicardial ablation, allows better contact of the ablator with less dispersion and greater precision in the ablation procedure. Finally, the management of complications, such as cardiac rupture, with a volume recovery system allows the patient to be “saved” through the accessory recovery of the blood, which will be re-infused for maintenance of acute volume and body oxygenation.

It is also worth noting that, in contrast to Impella, an *ad hoc* organization is required for the proposed strategy (e.g., a hybrid operating room or ventilator and cardiopulmonary bypass in the catheterization lab).

In summary, the MiECC III system has the advantages of allowing patient oxygenation and facilitating volume management compared with ECMO. However, the MiECC III system requires expertise and *systematic* organization, in addition to a more invasive procedure than the Impella device.

To the best of our knowledge, our experience is the first study to describe the successful application of the MiECC type III system during VT ablation.

## Conclusion

In conclusion, in this preliminary retrospective case series, given the aforementioned aspects, we believe that the MiECC type III system may represent an ideal support system during VT ablation. At present, our experience is still limited and should be regarded as a “how to do it” description and a “pilot.” Well-designed randomized trials are needed to support the use of these systems during VT ablation, and we suggest the adoption of only MiECC support to demonstrate a clinical advantage in patients receiving hemodynamic support compared to those without support.

## Data Availability

Data are available on request from the author.
